# Smart thermosensitive liposomes for effective solid tumor therapy and in vivo imaging

**DOI:** 10.1371/journal.pone.0185116

**Published:** 2017-09-21

**Authors:** Kevin Affram, Ofonime Udofot, Mandip Singh, Sunil Krishnan, Renee Reams, Jens Rosenberg, Edward Agyare

**Affiliations:** 1 College of Pharmacy and Pharmaceutical Sciences, Florida A & M University, Tallahassee, Florida, United States of America; 2 The University of Texas MD Anderson Cancer Center, Houston, Texas, United States of America; 3 The National High Magnetic Field Laboratory, Florida State University, Tallahassee, Florida, United States of America; University of South Alabama Mitchell Cancer Institute, UNITED STATES

## Abstract

In numerous studies, liposomes have been used to deliver anticancer drugs such as doxorubicin to local heat-triggered tumor. Here, we investigate: (i) the ability of thermosensitive liposomal nanoparticle (TSLnp) as a delivery system to deliver poorly membrane-permeable anticancer drug, gemcitabine (Gem) to solid pancreatic tumor with the aid of local mild hyperthermia and, (ii) the possibility of using gadolinium (Magnevist^®^) loaded-TSLnps (Gd-TSLnps) to increase magnetic resonance imaging (MRI) contrast in solid tumor. In this study, we developed and tested gemcitabine-loaded thermosensitive liposomal nanoparticles (Gem-TSLnps) and gadolinium-loaded thermosensitive liposomal nanoparticles (Gd-TSLnps) both in *in-vitro* and *in-vivo*. The TSLnps exhibited temperature-dependent release of Gem, at 40–42°C, 65% of Gem was released within 10 min, whereas < 23% Gem leakage occurred at 37°C after a period of 2 h. The pharmacokinetic parameters and tissue distribution of both Gem-TSLnps and Gd-TSLnps were significantly greater compared with free Gem and Gd, while Gem-TSLnps plasma clearance was reduced by 17-fold and that of Gd-TSLpns was decreased by 2-fold. Area under the plasma concentration time curve (AUC) of Gem-TSLnps (35.17± 0.04 μghr/mL) was significantly higher than that of free Gem (2.09 ± 0.01 μghr/mL) whereas, AUC of Gd-TSLnps was higher than free Gd by 3.9 fold high. TSLnps showed significant Gem accumulation in heated tumor relative to free Gem. Similar trend of increased Gd-TSLnps accumulation was observed in non-heated tumor compared to that of free Gd; however, no significant difference in MRI contrast enhancement between free Gd and Gd-TSLnps *ex-vivo* tumor images was observed. Despite Gem-TSLnps dose being half of free Gem dose, antitumor efficacy of Gem-TSLnps was comparable to that of free Gem(Gem-TSLnps 10 mg Gem/kg compared with free Gem 20 mg/kg). Overall, the findings suggest that TSLnps may be used to improve Gem delivery and enhance its antitumor activity. However, the formulation of Gd-TSLnp needs to be fully optimized to significantly enhance MRI contrast in tumor.

## Introduction

Pancreatic cancer (PCa) remains the fourth leading cause of cancer-associated deaths in the United States due to its high malignancy, poor prognosis and profound resistance to chemotherapeutic agents [1–4]. Non-availability of clinically important tumor biomarkers for early detection results in another setback of late stage diagnosis. Even though a few pancreatic ductal adenocarcinoma (PDAC) tumor markers have been identified, they lack specificity and provide sub-optimal sensitivity [5, 6]. Detection of tumors of 10 mm or less has been difficult though, MRI has proven to be promising in the diagnosis of early stage PDAC [7]. The difficulty in detecting tumors early has necessitated the development of highly sensitive agents for noninvasive diagnoses of PDAC at its earliest stages [5]. Conventional low molecular weight paramagnetic agents such as gadolinium chelates (Gd) have over the years found immense applications in tumor visualization and vascular imaging [8]; Nonetheless, this approach suffers from rapid extravasation into the extracellular compartment. This translates into a very narrow window for image acquisition that is unachievable in most situations. Consequently contrast enhancement is limited and blurred images are the result of the short *in vivo* half-lives [9, 10].

The therapeutic outcomes in the treatment of PCa have not improved significantly in many decades. Gemcitabine (Gem) is one of the anticancer drugs often used in the treatment of PCa, either as a single agent or in combination with other chemotherapeutic agents such as (leucovorin, fluorouracil, irinotecan and oxaliplatin, and) (FOLFIRINOX) [3, 11]. Among the major setbacks that have restricted the optimum effect of these drugs are chemoresistance, lack of tumor specificity and poor membrane permeability [3, 11, 12]. Limited penetration and resistance to therapy have been due to the tumor microenvironment that presents elevated intra-tumoral pressure which impedes the effective transport of drugs between the tumor and capillaries [13]. Therefore, exploitation of this tumor architecture and dysfunctional vasculature would promote the deposition of chemotherapeutic agents within the core of pancreatic solid tumor. Further, poor therapeutic outcomes have also been attributed to the systemic biochemical instability of the drugs [14]. Metabolism and rapid elimination of Gem significantly affect its bioavailability [15]. Cytidine deaminase is widely reported to rapidly convert Gem to 2’,2’- difluoro-deoxyuridine (dFdU) especially in the liver and to a lesser extent in plasma [16, 17]. Clinically, this leads to administration of high dose of Gem to achieve a therapeutic concentration which eventually causes severe side effects and hepatic toxicity [18, 19].

Conventional liposomes constitute one of the few delivery strategies that have resulted in approved products for clinical use in oncology [20]. Despite the success, there are limitations to therapeutic use of liposome-based formulations. These include variability in tumor uptake and accumulation of liposomes due to clinical heterogeneity in the enhanced permeability and retention (EPR) effect, poor tumor penetration of carrier and limited drug release at tumor site. However, external trigger-release liposomes such as thermosensitive liposomes have the potential to overcome these obstacles by providing triggered drug release under conditions of mild hyperthermia. Besides, thermosensitive liposomes can be designed to provide intravascular drug release or to release the drug following entry into tumor interstitium [20].

**Fig 1:**
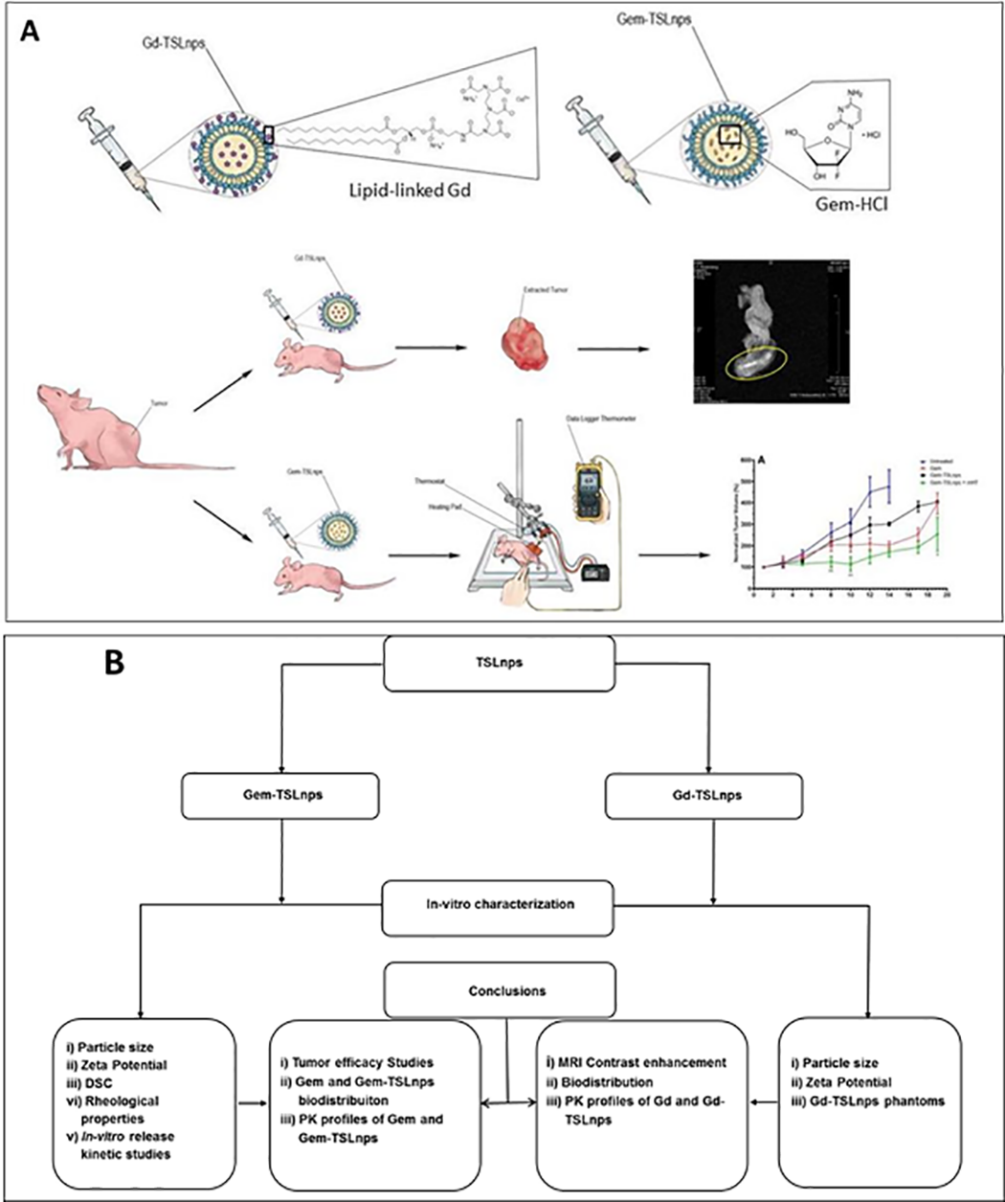
**(A)Tumor inhibition curve and ex-vivo tumor MR imaging**. Thermosensitive liposomal nanoparticles loaded-gadopentetic acid (Magnevist^®^, a gadolinium-based MRI contrast agent) was injected intraperitoneally and excised tumor was imaged and area circled yellow indicates a higher contrast area. Gd-TSLnps, and thermosensitive liposomal nanoparticles loaded gemcitabine, Gem-TSLnps were intravenously injected through the tail vein of mice. Mild heat (mHT) was applied to tumor site containing Gem-TSLnps and tumor growth inhibition determined, **(B) Flow chart summarizing the various studies.** The diagram depicts graphical representation of studies conducted in a coherent fashion.

In our previous publication, we demonstrated performance improvement of Gem loaded-thermosensitive liposomes in cytotoxicity studies and significant decline in *in-vitro* MiaPaCa-2 cells viability [21]. It is important to note that MiaPaCa-2 cells were chosen in that study because they have been found to be highly tumorigenic [22] and resistant to gemcitabine treatment [23]. In the present study, we developed a novel TSLnp capable of increasing antitumor efficacy of Gem; however, Gd-TSLnp could not produce significant MRI contrast in *ex-vivo* tumor (Fig 1A and 1B).

Overall, our studies demonstrated that TSLnps: (i) improved antitumor efficacy of Gem, (ii) improved pharmacokinetic profiles of Gem and Gd (iii) increased distribution of Gem and Gd in tissues and organs and, (iv) could not significantly enhance Gd MRI contrast.

## Materials and methods

Dipalmitoyl phosphatidylcholine (DPPC),1-myristoyl-2-palmitoyl-sn-glycero-3-phosphocholine (MPPC), 1,2-distearoyl-sn-glycero-3-phosphoethanolamine-N-[amino(polyethylene glycol) 2000 (DSPE—PEG_2000_) and 1,2-dipalmitoyl-sn-glycero-3-phospoethanolamine-N-diethylenetriaminepentaacetic acid (gadolinium salt) Gd-DSPE were purchased from Avanti Polar Lipids (Alabaster, AL). Gadopentate dimeglumine (Magnevist^®^) was purchased from Bayer HealthCare Pharmaceuticals Inc. (Wayne, NJ). MiaPaCa-2 cell line was bought from American Type Culture Collection (ATCC) (Manassas, VA). All solvents used were of analytical grade.

### Formulation preparation

#### Gadolinium entrapped liposomal nanoparticles (Gd-TSLnps)

Gd-TSLnps was prepared according modifications from previous methods from our other publications [8, 24, 25]. Briefly, different lipids were weighed per the molar ratio of 70:5:20:4 for DPPC, MPPC, Gd-DSPE and DSPE-PEG_2000_, respectively. These lipids were dissolved in chloroform, which subsequently was removed using a dry stream of nitrogen gas followed by drying under vacuum to form a thin film. The thin film was hydrated with 2 ml phosphate-buffered saline (PBS) of 150 mM Gd and extruded through 200 and 100 nm polycarbonate membranes sequentially, ensuring that the temperature was above 60°C to obtain uniform sizes. The Gd entrapped liposome suspension was centrifuged at 6000 rpm for 5 min using a vivaspin^®^ (MWCO 30kDa) to remove free Gd.

#### Gemcitabine entrapped liposomal nanoparticles (Gem-TSLnps)

Gem-TSLnps was prepared in a similar fashion to Gd-TSLnps preparation. Briefly, DPPC, MPPC and DSPE-PEG_2000_ were weighed in the molar ratio (90:10:4) [26] and dissolved in chloroform to obtained a homogenous solution. As formed above, a dried thin film was hydrated with 2.0 ml of 10 mM Gem in PBS at a temperature of about 60°C and extruded 15 times through a 200 nm polycarbonate sandwiched between two filter supports. The formed liposomes were collected into glass vials and dialyzed using a dialysis bag (MWCO 12–14 kDa) against phosphate buffer (pH = 7.4) overnight at room temperature to remove free Gem.

### Particle size and zeta potential determination

The size and charge distribution of Gem-TSLnps and Gd-TSLnps were determined by measuring the mean hydrodynamic particle size and zeta potential of Gem-TSLnps and Gd-TSLnps via dynamic laser light scattering using the particle size analyzer. The instrument was first calibrated with reference or standard solutions (90, 200 and 400 nm particle size submicron latex solutions) according to the manufacturer’s instructions. Solutions of various formulation samples were prepared by adding 50 μL of each sample to 5 mL of distilled water, vortexed for 5s to yield a uniform suspension. An aliquot of each solution was used to determine the particle size and zeta potential. Measurements were done in triplicate.

#### Measurement of rheological properties of blank TSLnps

One milliliter (1.0 ml) of blank TSLnps was prepared similar to the preparation of Gem-TSLnps. For this formulation, a total weight of 50 mg of lipids (DPPC, MPPC and DSPE-PEG_2000_) was weighed, dissolved in chloroform and chloroform removed with nitrogen gas. Residual chloroform was eliminated by drying in a vacuum chamber. The thin film deposited in the inner wall of the glass vial was hydrated with 1.0 mL of PBS, and the final lipid concentration was 50 mg/ml. Rheological measurements (AR 1500ex, New Castle, DE) were performed with a cone diameter of 20.0 mm and cone angle of 1.0^o^. Rheological measurements were performed at room temperature (28.0°C), 37 ± 1.0°C and 42 ± 1.0°C at a loading gap of 1000 μm. The data was fitted in the power law model as shown below and was used to determine the rheological properties of the TSLnp suspension [27]:

$$\sigma =k\gamma^{n}$$ (1)

Taking the logarithm of both sides for Eq (1),

logσ=logk+nlogγ (2)

where σ is the shear stress, k is the consistency coefficient, γ is the shear rate and n is the flow behavior index. A plot of log η against log γ was used to determine whether the formulation was shear thinning or shear thickening.

### *In vitro* release kinetic studies of Gem-TSLnps

The cumulative release of Gem from the heat sensitive liposome nanoparticles was performed at 37 and 42°C. Twenty milligrams (20.0 mg) of lyophilized Gem-TSLnps was suspended in 1.0 ml of PBS (pH = 7.4), transferred into a dialysis bag (MWCO = 3,500) and incubated in 5 ml of PBS pre-equilibrated to temperature (37 or 42°C). One milliliter (1.0 ml) was collected at selected time points and replaced with 1.0 ml of fresh PBS. Collected samples were analyzed by HPLC–UV/Vis spectrophotometry to determine the amount of Gem released at different time points.

#### Mathematical models to determine the release mechanism

The release kinetics of Gem from the heat sensitive liposomes was investigated to predict the possible mechanism of release using mathematical models. The release order was determined using zero order (Eq 1) and first order kinetic model as shown below.

$$C=C_{o}+K_{o}t$$ (3)

$$Log\;C=Log\;C_{o}-\frac{K_{1}t}{2.303}$$ (4)

where C_o_ is the initial amount of drug, C is the % cumulative Gem released (zero order) or remaining (first order) at time “t” and K_o_ is zero order release constant and K_1_ is the first order release constant [28].

A Higuchi model was used determine whether the release mechanism follows Fickian diffusion as shown below [29]:

$$C=C_{o}+K_{H}t^{1/2}$$ (5)

where C is the % cumulative Gem release at time, t and K_H_ is the Higuchi constant.

The Korsmeyer-Peppas model, which is used to describe the release from a polymeric system, was used to model ≤ 60% of the cumulative drug release data.

$$C=K_{KP}t^{n}$$ (6)

where C is the % cumulative Gem release, K_KP_ is the Korsmeyer-Peppas constant and n is release exponent, which is used to describe the release mechanism [30, 31].

A Hixson-Crowell model, which describes drug release by dissolution and change in carrier surface area and diameter, was applied [30]:

$$C_{o}^{1/3}-C^{1/3}=K_{HC}\;t$$ (7)

where C is the drug % remaining in matrix at time, t and K_HC_ is the Hixson-Crowell constant.

### Determination of phase transition temperature (T_m_) for TSLnps

TSLnp was prepared in PBS as described in the formulation of Gem-TSL. Herein, the liposomes were prepared with the exclusion of Gem thus blank liposomes. Similarly, 100 mg of DPPC was dissolved in chloroform and dried under nitrogen gas, and the residual chloroform removed using in a vacuum chamber. The dried lipid was hydrated with 500 μL of PBS. The Differential scanning calorimeter (DSC Q100, TA instruments, New Castle, DE) was used to determine the phase transition temperatures, T_m_ of DPPC and TSLnps. Briefly, the instrument was initially equilibrated for about 45 min. After, a hermetic aluminum pan was filled with 20–30 μL of DPPC or TSLnps suspension, carefully covered with aluminum lid and sealed tightly. Measurement was conducted at 5.0°C/min with temperature max set at 70°C. Both DPPC and TSLnps analyses were conducted in a pure dry nitrogen atmosphere.

### Preparation and MRI of nanoparticle phantoms

Various dilutions of Gd-TSLnps with distilled water in ratio of 1:1, 1:5, 1:10 and 1:100 were made. Each dilution was then mixed with 1% agarose in the ratio 1:1 and carefully injected into microcapillary tube with open ends by suction and allowed to solidify. The ends of each tube were sealed using wax. A control phantom also was prepared by mixing equal volume of distilled water, and 1% agarose in a ratio of 1:1 and allowed to solidify. Samples were stored at 4°C prior to MRI [32]. All MRI was performed on a 21.1 T (900 MHz) vertical magnet located at the National High Magnetic Field Laboratory (NHMFL)[33]. The magnet is equipped with a Bruker Avance III spectrometer and ParaVision 5.1 acquisition software (BioSpinCorp, Billerca, MA). A 64-mm inner diameter high performance gradient (Resonance Research Inc, MA) was used together with a 10-mm birdcage radio frequency (RF) coil. All nanoparticle phantoms were imaged in unison. Measurements were set up to quantify R_1_ (1/T_1_) and R_2_ (1/T_2_) relaxation rate. Samples were acquired with 100x100 matrix, resulting in 100x100 μm in plane resolution, and one slice with a thickness 1-mm was used. Measurements were performed with a spin echo (SE) sequence using nine incrementing repetition times (TR = 26–15000 ms) and 16 incrementing echo times (TE = 10–160 ms) for R_1_ and R_2_ respectively. R_2_ relaxation rates were extracted by plotting signal intensities from regions of interest (ROIs) covering the samples versus TE using a single exponential decay function. R_1_ relaxation rates were extracted using signal intensities from same ROIs vs TR, and plotted with a single exponential growth function with baseline adjustments. By plotting the relaxation rate as a function Gd concentration, the relaxivity (mM^-1^s^-1^) of the sample could be calculated. All plots and non-linear regressions were done with SigmaPlot 7.101 (SPSS Inc, Chicago IL).

### Animals

Female athymic nude (Nu/Nu) mice were obtained from The Jackson Laboratory (Bar Harbor, ME) at 6 to 8 weeks of age. The mice were housed in a virus-free, indoor, light- and temperature controlled barrier environment, and were provided *ad libitum* access to food and water. All procedures with mice were in strict accordance with the National Institutes of Health Guide for the Care and Use of Laboratory Animals and approved by the Florida A & M University Animal Care and Use Committee.

#### Subcutaneous tumor studies

MiaPaCa-2 cells (5 × 10^6^ cells injected per mouse) were used to establish subcutaneous tumors on the right flanks of mice. Treatment began when tumors reached a palpable size (~ 40–70 mm^3^) with five mice per group. All the four groups (control, Gem, Gem-TSLnps and Gem-TSLnps followed by mild hyperthermia (mHT)) were administered with normal saline, Gem or Gem-TSLnps by intraperitoneal injection (i.p) every other day for two weeks (total of eight injections). IP-injection was chosen as a route to deliver the drug/formulation because of the following reasons: i) to prevent vascular damage due to the repeated injections (4 injections per week) because of the difficulty in tracing vein in mouse tail, ii) to avoid severe stress on animals, which needed to be restrained if not anesthetized during injection, and iii) It is less tedious compared to IV injection and it is the most common and widely used mode of drug administration for small lab animals. Gem was given at a dose of 20 mg/kg while Gem-TSLnps was administered at a dose of 10 mg/kg (Gem equivalent) to Gem-TSLnps or Gem-TSLnps + mHT treated group. The different dose rates were due to viscosity adjustment of Gem-TSLnps solution with PBS (1x) to that of free Gem solution, resulting in reduced concentration of Gem in TSLnps by half. Per our institutional animal care and use (IACUC) approved protocol, equal volumes (0.2 mL per 24g body weight) of Gem-TSLnps and free Gem solutions were to deliver to mice per injection. Therefore, the dose rates were different for free Gem and Gem-TSLnps as of results viscosity adjustment and equal delivery volume. After two weeks, Gem-TSLnps + mHT mice were exposed to mHT (41°C±1) at the tumor area for period of 10 min.

Mild hyperthermia treatment: Mice were subjected to localized form of mHT treatment. In the application of mHT, we employed a specially designed heating system equipped with a thermostat, a heating pad and thermometers (temperature sensors). The room was preheated to 27 ± 2 ^o^C prior to mHT treatment. The heating system was set at 41± 2°C and heating pad adjusted perpendicularly over the skin surface of the tumor in such a way that the skin surface temperature of the tumor was maintained at 41± 2°C. Once the temperature stabilized at 41± 2°C, thermometers were placed both on the skin surface and in the core of the tumor to measure temperatures. In all, it took 7 minutes to reach a steady temperature of 41± 2°C at skin surface of tumor and 40 ± 1°C in the core and sustained these temperatures for extra 3 minutes. During the 3 minutes period, temperatures were measured at different areas on the skin surface and core of the tumor. The heating pad was automatically switched–off when skin surface temperature of the tumor reached 43°C for a period of 60 seconds. Heating resumed again when the skin surface temperature dropped to 40°C. Overall, mHT treatment was initiated when the tumor volume reached a range of 40–70 mm^3^. Tumor measurements were performed using a pair of digital vernier calipers performed every other day for 19 days. Mice with tumor size 20 x 20 mm or larger were sacrificed to prevent pain and suffering. Tumor volume was determined using the equation below:

$$Tumor\;volume\;({mm}^{3})=\frac{L*W^{2}}{2}$$ (8)

where L is the length (mm) of the long axis, W is the length (mm) of the short axis.

A Kaplan-Meier (K-M) estimate was determined by recording the number of animals that were censored over time in each group study. Parameter for censoring was based on larger tumor sizes (≥ 20 × 20 mm) which subjected the animals to unbearable pain. Surviving animals were also recorded accordingly and a survival curve plotted using GraphPad Prism software (GraphPad Software, Inc., La Jolla, CA, USA).

#### MR imaging of *ex vivo* mouse tumor

Mice bearing-MiaPaCa-2 tumors in right hind limbs were grouped into controls, Gd (from Magnevist^®^) and Gd-TSLnps. Normal saline (control), Gd (4 mg/kg) and Gd-TSLnps (4 mg/kg Gd content) injections were administered as previously described; however, the tumor-bearing mice were not exposed to mHT. The rational was to confine Gd within the tumor and limit the release and diffusion of Gd back to the systemic circulation and to other tissues. Mice were sacrificed at 30, 60 and 90 min time points, and their tumors extracted, rinsed with PBS and finally stored in 4% paraformaldehyde. Prior to MRI, the tumor was rinsed in PBS for 24 h followed by placement in a 10-mm NMR tube and submersion in Flourinert (3M Center, St. Paul, MN), a perflourinated liquid with no background ^1^H MRI signal. Using the 21.1 T (900 MHz) magnet and a 10-mm RF coil, a T_1_-weighted spin echo sequence was employed. Acquisitions were made with a 50x50 μm in-plane resolution and 0.75 mm slice thickness. TR was incremented between 200–500 ms using a constant TE of 15 ms. T_1_ maps were generated with ParaVision 5.1.

#### Pharmacokinetics (PK) and bio-distribution

In another study, mice were grouped into controls, Gd and Gd-TSLnps, and were injected intraperitoneally with normal saline, Gd or Gd-TSLnps, respectively, and as previously described. Aliquots of blood samples (50 μL) at different time points (5, 10, 15, 30, 60, 120, 240, 360, 720 and 1440 min) and various tissues including tumors were excised after 24 hours. Blood samples and tissues were pretreated with aqua regia (HNO_3_: HCl) in a volume ratio 1:3 and allowed to stand overnight in a fume hood. The pretreated samples were further diluted with 2% nitric acid (70%, v/v) in distilled water, centrifuged at 4,000 rpm for 10 min and finally filtered to remove any debris. The final sample solutions were analyzed by inductively coupled plasma mass spectrometry (ICP-MS) to determine the quantity of Gd in each sample solution.

In a related study, bio-distribution and PK of Gem and Gem-TSLnps were conducted in a similar manner as described above, except that mice in Gem and Gem-TSLnps groups were injected with bolus dose of 20 mg/kg of Gem or Gem-TSLnps (Gem equivalent to 10 mg/kg). Herein, equal doses (Gem) were administered to mice in the PK studies to ascertain the bioavailability (area under the concentration curve, AUC measurement). Blood samples collected at each time point was treated with 1 ml of 15% isopropyl alcohol in ethyl acetate (extraction solvent), vortexed for 20–30 s and centrifuged at 3,000 rpm for 15 min. Tissues were homogenized in 1 ml 15% isopropyl alcohol in ethyl acetate with the volume increased to 2.5 ml with addition of extraction solvent. The solution was vortexed and centrifuged at 6000 rpm for 15 min [34]. The supernatant of both blood and tissues samples were vaporized in a water bath and placed in a vacuum chamber overnight to remove residual solvent. The dried samples were reconstituted with 500 μL of mobile phase (5% acetonitrile in 10 mM dihydrogen phosphate buffer, pH adjusted to 3) and centrifuged. The supernatants were filtered and, the filtrates of either the blood or tissue samples were analyzed for the presence of Gem using for HPLC-UV [35].

The Gem or Gd plasma concentration profile after a single i.v. bolus dose was best described by a biexponential disposition function below:

$$Cp=A*e^{-\alpha *t}+B*e^{-\beta *t}$$ (9)

where Cp is the drug plasma concentration at time t, A is initial plasma concentration in the distribution phase, α is first order transfer rate constant of the distribution phase (hr^-1^), B is initial plasma concentration in the elimination phase, β is first order transfer rate constant of the elimination phase (h^-1^) and t is time (h). Pharmacokinetic parameters were estimated using software Pharmacokinetic Solutions 2.0 (PK Solutions). Secondary parameters such as volume of distribution compartment (V_d_), clearance (CL), half-life (t_1/2_), elimination rate constant (K_el_), AUC curve, and mean residence time (MRT) distribution rate constants from central to peripheral compartment and from peripheral to central compartment (K_12_ and K_12_, respectively) were also determined.

#### HPLC analysis

Gem analysis was performed according to method described by Lanz and colleagues with minor modifications [35]. Briefly, Gem analysis was performed using a chromatographic system, which consisted of a HPLC (Waters Corporation, Milford, MA) equipped with an auto-sampler, photo diode array (2998 UV/Vis) detector and pumps. Separation was performed using a reverse phase column (ZORBEX SB–C18 4.6 x 250 mm, 5 μm). A flow rate of 1.0 ml/min and injection volume 20μl at ambient temperature were maintained while detection was performed at 268 nm. Prior to analysis, reverse phase column was equilibrated with mobile phase made up of 5% acetonitrile in 10 mM dihydrogen phosphate buffer, pH adjusted to 3 with trifluoroacetic acid (TFA). An isocratic elution was performed throughout the entire analysis including internal standards.

A calibration curve was prepared using Gem standard solutions with concentration range of 0.063–2.0 μg/mL. A plot of the peak areas as a function of Gem concentration was plotted and the linear equation of the calibration curve given as y = mx + c was determined, where y is the peak area, m is the slope, x is the concentration of Gem and c is the y—intercept was. Supernatants from controls were spiked with aliquots of 0.5 μg/ml of Gem. Recovery of Gem in supernatant from blood and tissues was performed by comparing peak areas of controls spiked with known amounts of Gem [35].

### Statistical analysis

The difference between Gem and Gem-TSLnps as well as Gd and Gd-TSLnps treatment groups were analyzed using paired Student’s t-test and considered significant at p < 0.05. All experiments were performed at least in triplicate and analyzed using GraphPad Prism software (GraphPad Software, Inc., La Jolla, CA, USA).

## Results

### Characterization of TSLnps

Sterically stabilized Gem-TSLnps or Gd-TSLnps was formulated by adding DSPE-PEG_2000_ for improved stability and increased *in vivo* half-life compared with free Gem or Gd. The strategy was to prepare two TSLnps delivery systems with one encapsulated with Gem and other encapsulated only with Gd. This strategy allows for higher payload of Gem or Gd in TSLnps.

#### Particle size and zeta potential of TSLnp

Two types of liposomal nanoparticles, namely Gem-TSLnps and Gd-TSLnps were prepared through the film hydration/extrusion method. The hydrodynamic diameter, polydispersity index (PI) and net surface charge of Gd-TSLnps were 170.4 ± 3.12 nm, 0.17 ± 0.03 and 2.28 ± 0.19 mV, respectively. While Gem-TSLnps yielded slightly larger particle size of 216.10 ± 0.57 nm, PI = 0.13 ± 0.026 and almost neutral surface charge of -0.047 ± 0.002 mV (Table 1).

**Table 1:** Characterization of Gd-TSLnps and Gem-TSLnps.

Formulation	Mean Particle size (nm)	Mean Zeta Potential (mV)	Polydispersity Index (P.I)	Entrapment Efficiency (%)
Gd-TSLnps	170.4 ± 3.12	2.28 ± 0.19	0.17± 0.032	62.6 ± 8.02
Gem-TSLnps	216.10 ± 0.57	-0.047 ± 0.002	0.13 ± 0.026	41.1 ± 2.02

Data represents mean ± SD, n = 3

### Viscosity of TSLnps

**Fig 2:**
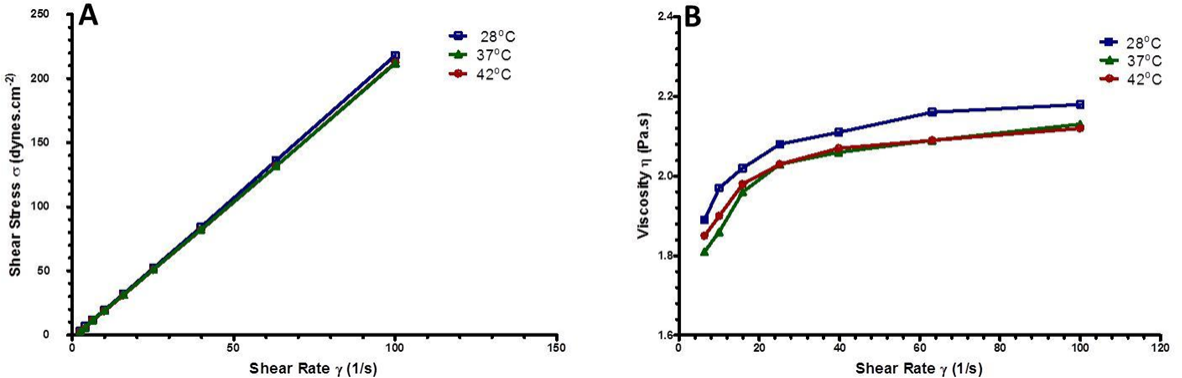
The rheological profile of TSLnps. (A) Flow curve of TSLnps at room temperature (28.0°C), 37°C and 42°C with a linear increase shear stress with increasing shear rates; (B) Viscosity of TSLnps as a function of shear rate at room temperature (28 ^o^C), 37°C and 42°C with insignificant effect of shear rate on viscosity.

To determine whether viscosity difference may impact stability and mobility of TSLnps, we conducted viscosity studies on TSLnps solution at 28, 37 (normal human physiological temperature) and 42°C, and the results are presented in Fig 2. The plot of shear stress versus shear rate (Fig 2A) at a given temperature shows a linear relationship with a constant slope. No significant difference (p > 0.05) among the slopes was observed. A plot of viscosity (the slope) against shear rate (Fig 2B) depicted a slight rise in viscosity and thereafter became constant independent of the shear rate. In particular, no significant difference (p > 0.05) was observed between viscosity and increasing temperature (Table 2).

**Table 2:** Rheological properties of TSLnps at varying temperatures.

Temperature	Viscosities (cps)	Consistency coefficient (k)	Flow behavior index (n)
28°C	219.89 ± 2.23	1.48 ± 0.01	1.10 ± 0.02
37°C	213.92 ± 0.93	1.28 ± 0.03	1.13 ± 0.01
42°C	214.80 ± 1.21	1.13 ± 0.01	1.16 ± 0.04

Data represents mean ± SD, n = 3.

### Thermotropic phase behavior of TSLnps

**Fig 3:**
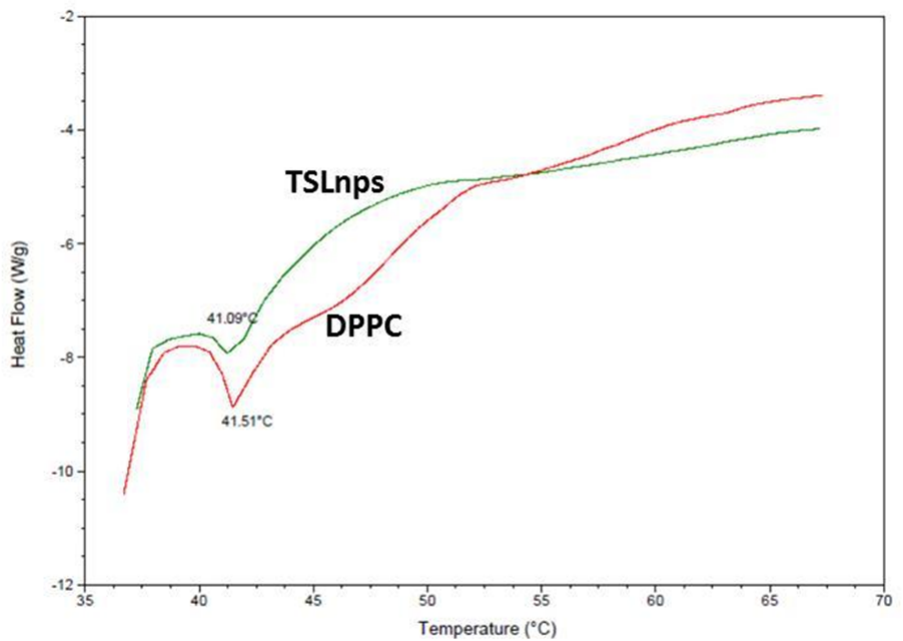
Differential scanning calorimetry (DSC). Thermogram analysis of DPPC and TLSnps.

The DSC data in Fig 3 displayed a broad transformation curve (endothermic transition) of TSLnp at 41.09°C; however, pure DPPC showed a sharp peak around 41.51°C close to what has been reported by others [36]. The inclusion of MPPC and DSPE-PEG_2000_ in formulating TSLnp is reported to influence the shifting of transition phase temperature slightly from T_m_ = 41.51°C from T_m_ = 41.09 ^o^C with broader trough compared to that of pure DPPC alone as shown in Fig 3 [37]. Put together, the broad peak and slightly lower phase transition temperature of TSLnps indicates that TSLnps may have different thermal behavior compared with DPPC.

### *In vitro* release kinetics of Gem-TSLnps

**Fig 4:**
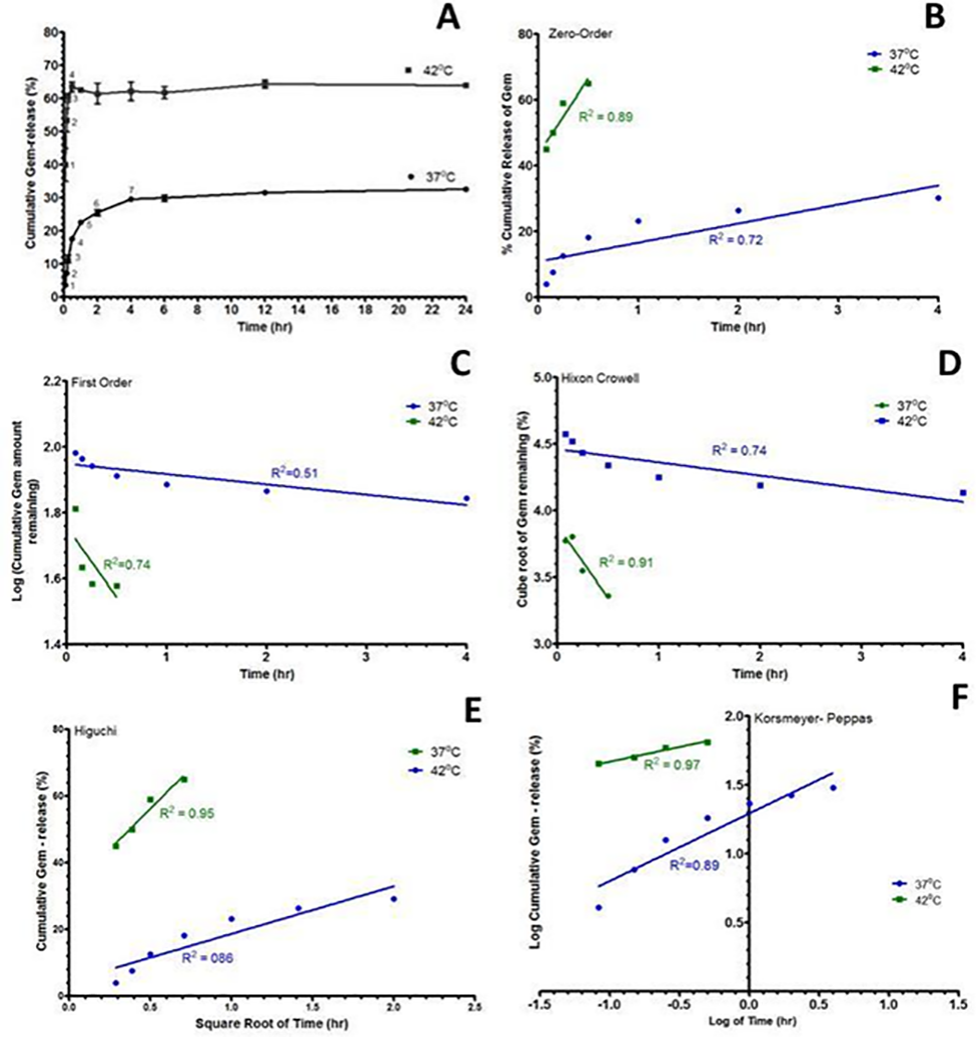
In-vitro release profile and kinetics of gemcitabine loaded thermosensitive liposomal nanoparticles (Gem-TSLnps). (A) In vitro cumulative release profile of Gem as a function of time in phosphate buffer saline (PBS) at 37± 0.5 ^o^C and 42 ± 0.3 ^o^C; (B) Zero order kinetics; (C) First order kinetics; (D) Higuchi modeling; (E) Korsemeyer-Peppas modeling; (F) Hixson-Crowell modelling. In-vitro release kinetic study on Gd-TSLnps was not conducted because our previous study on invitro Gd showed negligible amount of Gd release [32].

The release kinetics was studied at 37 and 42°C. The release pattern of Gem showed a slow release (29.6%) within the first 4 h at 37°C (indicated by a point numbered 7 on the curved graph @37 ^o^C, Fig 4A) whereas at 42°C, rapid Gem release (63.5%) occurred in the first 24 min (indicated by a point numbered 4 on the curved graph at 42°C, Fig 4A). Thereafter, both 37 and 42°C curves attained fairly constant release behavior independent of time for next 22 h. There was a significant difference in the cumulative release of Gem between 37 and 42°C (p>0.01).

To analyze the mechanisms of Gem release-rate kinetics at 37 and 42 ^o^C, the *in vitro* release profiles were plotted in various kinetic models: zero-order release (Fig 4B), first-order release (Fig 4C), Hixson-Crowell model (Fig 4D), Higuchi model (Fig 4E) and Korsmeyer-Peppas model (Fig 4F). For this purpose, the linear portions of curved graphs at 37 and 42°C were used to model Gem release kinetics as shown. The coefficient of determination (R^2^) of the release kinetic models was calculated from the following plots; zero-order, first-order, Hixson-Crowell, Higuchi and Korsmeyer-Peppas models at 37 and 42°C, Fig 4A–4F. Among these models, the best linearity was found in Korsemeyer-Peppas plot (R^2^ = 0.97 at 42°C) with a Fickian release exponent (n = 0.22). R^2^ > 0.95 was used to determine the best fit of the model.

### Gd-TSLnp phantoms

**Fig 5:**
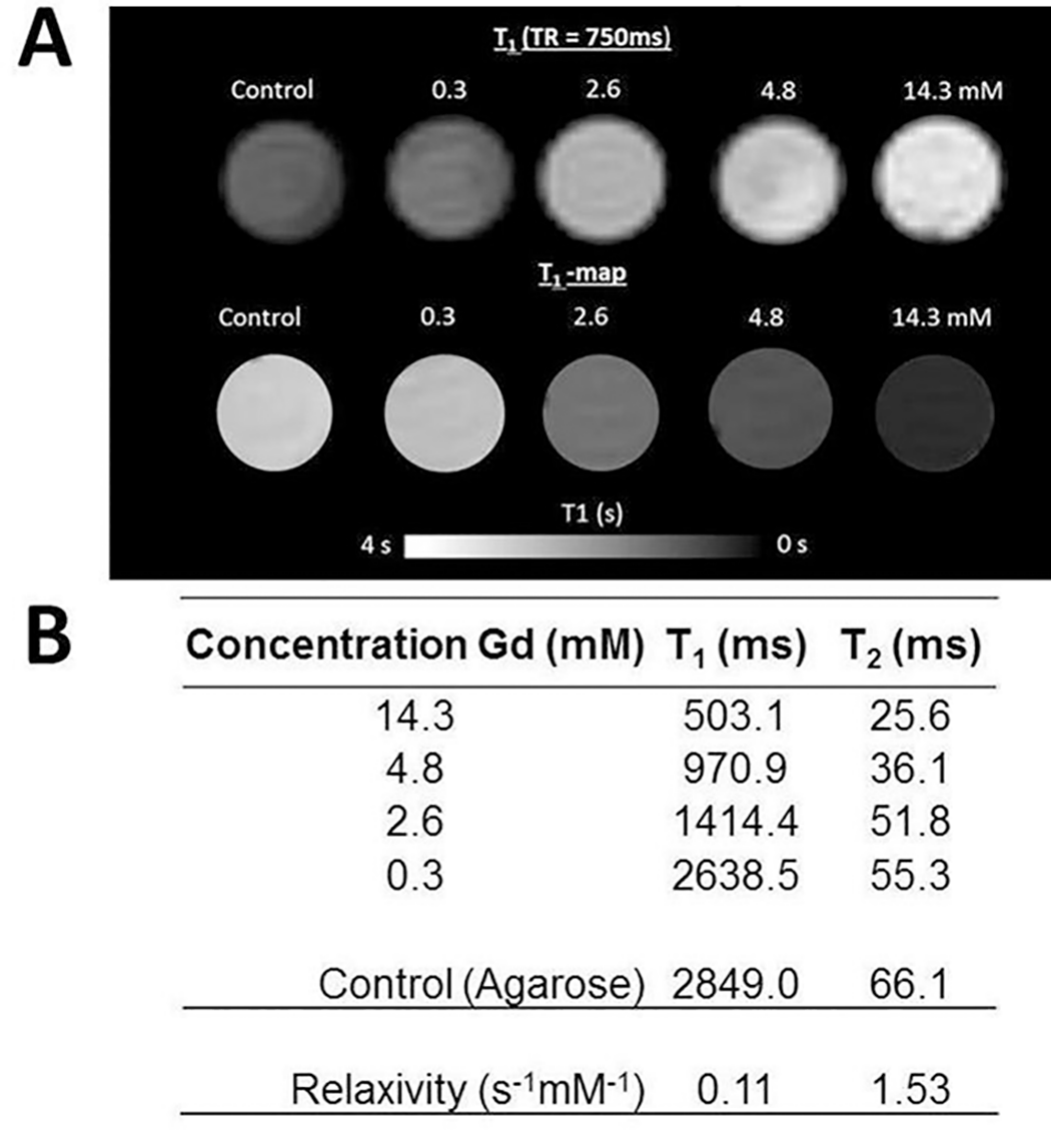
Phantom images. (A): The top row show T_1_ weighted images with increasing signal with increased Gd concentration at TR = 750 ms. The bottom row show the corresponding T_1_ maps for each respective Gd concentration: (B) T_1_ and T_2_ values of varying concentrations of Gd only and Gd in TSLnps with the corresponding relaxivity values.

To track TSLnps noninvasively in pancreatic tumor and monitor their tissue biodistribution with MRI, Gd-TSLnps were formulated by encapsulating TSLnps with a Gd complex. Different concentrations of Gd were incorporated into TSLnps to determine the Gd molar concentration that would exhibit the highest contrast. A close examination of T_1_-weighted images showed an increased contrast with increasing Gd concentration (Fig 5A), which is mirrored by a corresponding decreased in T_1_ (ms) and T_2_ (ms) values (Fig 5B). In all, TSLnps with Gd concentration of 14.3 mM exhibited the highest contrast, while TSLnp with 0.3 mM Gd showed the weakest contrast.

### MR imaging of *ex vivo* tumor

**Fig 6:**
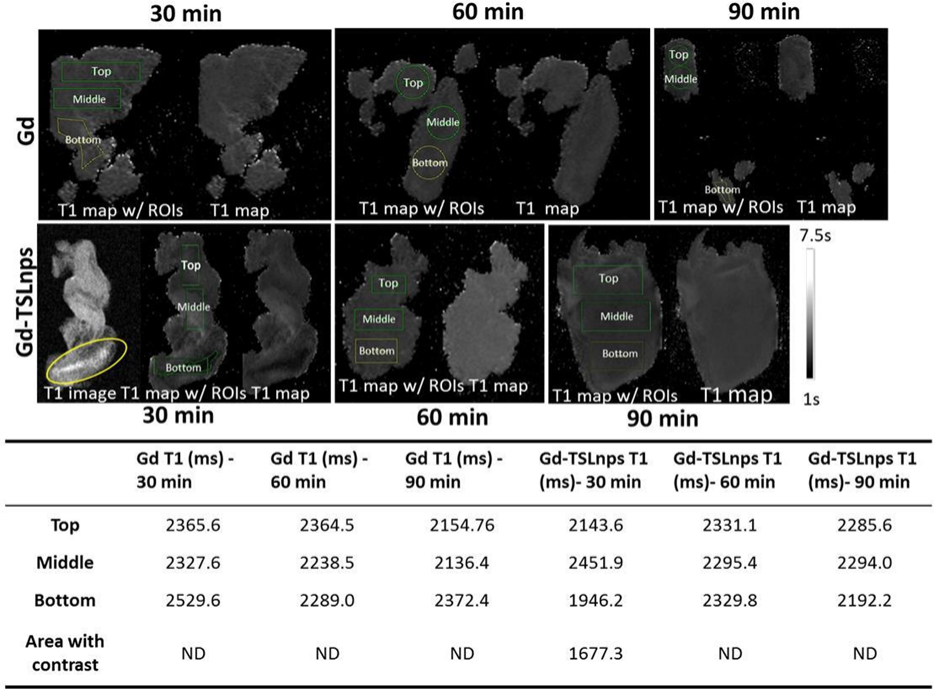
*Ex vivo* MRI scan. T_1_ maps of *ex vivo* tumors acquired at 30, 60 and 90 min after i.p. injection of free Gd and Gd-TSLnps. On the left of each sample, the ROI placement for T_1_ extraction is shown. The right full T_1_ map is shown. The 30-min Gd-TSLnps sample showing a region with increased T_1_ contrast also has the magnitude image included. **ND = not detected**

To determine whether the accumulated amount of Gd-TSLnps in tumor was sufficient for visualization using MRI, sections of *ex vivo* tumor were imaged at 21.1 T. Tumor excised at 30 min post injection of Gd-TSLnps showed a bright contrast (circled yellow, Fig 6) whereas images acquired at 60 and 90 min post injection in *ex vivo* tumors showed no visible contrast. A similar trend to that of 60 and 90 min post injection of Gd-TSLnps was observed when *ex vivo* tumors of mice injected with equivalent dose of Gd showed no visible contrast at 30, 60 or 90 min time point (Fig 6). Mapping of the different regions of the excised tumors provided approximately the similar T_1_ relaxation coefficients (ms) except for the 30 min *ex vivo* tumor, which exhibited a shorter T_1_ relaxation (T_1_ = 1.68 s) indicating the presence of Gd-TSLnps (Fig 6, yellow circle).

Although no visible contrast was observed for the other predefined time points for both Gd and Gd-TSLnps, it did not rule out the presence of Gd. There is the possibility that the concentration of Gd was below detection limit. Arguably, high payload of Gd in liposomal nanoparticles would significantly improve the window of opportunity for MRI of obscure and early tumors conspicuously (Fig 6).

### *In vivo* antitumor efficacy

**Fig 7:**
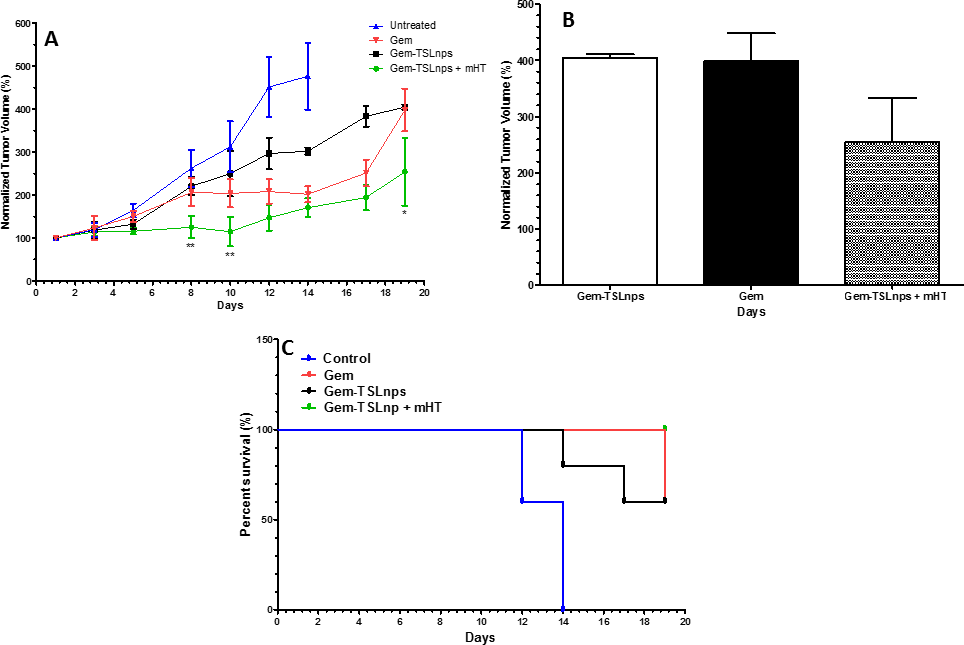
Activity of Gem-TSLnps in MiaPaCa-2 tumor-bearing mice. **(A)** Tumor growth curves showing greater growth inhibition for Gem-TSLnps + mHT (Gem dose, 10 mg/kg) compared with Gem only (Gem dose, 20 mg/kg); **(B)** End points of normalized tumor volume (on 19^th^ day) of Gem only, Gem-TSLnps and Gem-TSLnps + mHT treated groups. **(C). Kaplan-Meier survival curve for tumor-bearing mice**. Mice grouped into: i) Control ii) free Gem iii) Gem-TSLnps iv) Gem-TSLnps + mHT were treated and survival (%) plotted against time. The tick marks represent time a mouse was terminally censored. p* < 0.05, p**<0.01, where * or ** is comparison of free Gem and Gem-TSLnps + mHT treated groups. Data expressed as mean ± SEM, (n = 5 per group).

The anti-tumor activities of Gem and Gem-TSLnps with and without heat were evaluated, and the results shown in Fig 7A. It was evident that the tumor growth for control mice increased rapidly within 14 days while the tumor growth of mice treated with Gem, Gem-TSLnps and Gem-TSLnps + mHT were significantly inhibited compared with that of the control group. Furthermore, there was no significant difference between the tumor volumes of Gem and Gem-TSLnps treated groups on the 19^th^ day as shown in Fig 7B. Among the three treated groups, anti-tumor inhibition of Gem-TSLnps + mHT (at Gem equivalent dose of 10 mg/kg) treated group was significantly increased when compared with Gem (at Gem dose of 20 mg/kg) and Gem-TSLnps (at Gem equivalent dose of 10 mg/kg), implying that the anti-tumor activity of Gem was improved through the delivery system of TSLnp combined with mHT. A Kaplan Meier survival curve was used to monitor the survival of the animals in the control and treatment groups as well. As observed in Fig 7C, 100% of the animals in Gem-TSLnps + mHT survived entire study period (19 days) compared to 60% survival for Gem treatment group. Similarly, 60% of the animals survived in the group that received Gem-TSLnps without mHT. On the other hand, only 60% of the mice in the control group survived by the 12^th^ day with no live (100% censored) by day 14.

### Plasma pharmacokinetics and bio-distribution TSLnps

To provide improved protection and/or reduced renal clearance for short half-life or easily degraded Gem and provide a prolonged pharmacological effect, pharmacokinetic profiles and biodistribution of Gem and Gem-TSLnps were monitored to properly describe the behavior of Gem or Gem-TSLnps with combined with mHT *in vivo*.

#### Gem and Gem-TSLnps

**Fig 8:**
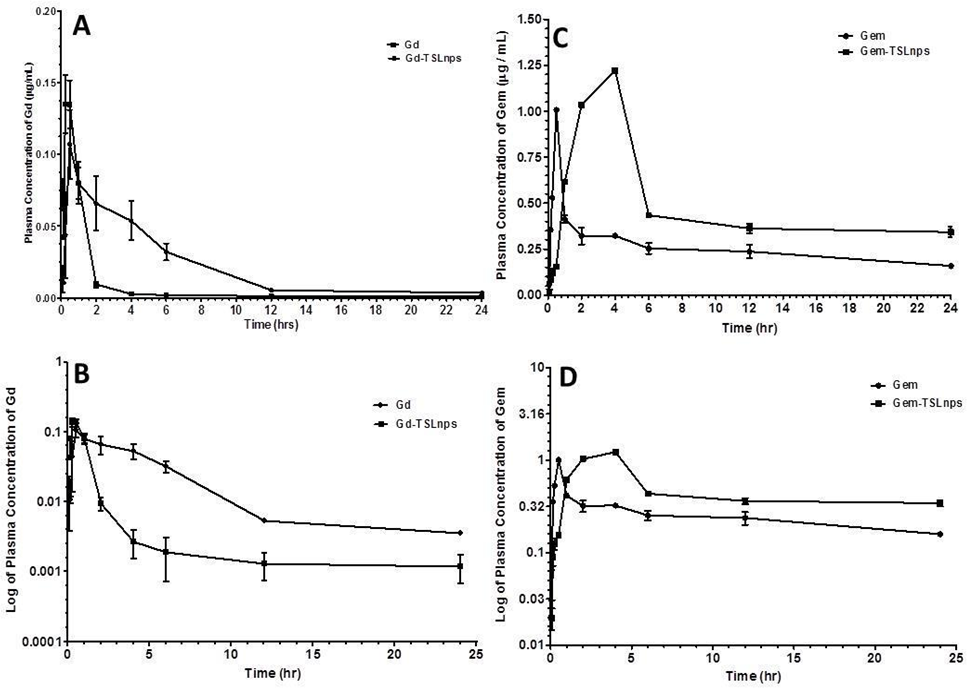
Plasma concentration-time curves of measured Gd-TSLnps and Gem-TSLnps. A) Plasma concentration of Gd only and Gd-TSLnps as a function of time. B) Plasma concentration of Gem only and Gem-TSLnps as a function of time. C) Log plasma concentration of Gd only and Gd-TSLnps against time. D) Log plasma concentration of Gem only and Gem-TSLnps against time. Concentration of Gd (Magnevist^®^) was measured by inductively coupled plasma mass spectrometry (ICP-MS), while concentration of Gem was measured by HPLC. Data represent mean ± SD, (n = 5 per group).

After i.p. single dose of Gem (20 mg/kg) injection and Gem-TSLnps (at Gem dose equivalent of 10 mg/kg), plasma concentration-time curve showed a biphasic behavior with a clear distinct distribution and elimination phases. Also, the Gem curve tends to have a faster absorption rate while Gem-TSLnps curve seems to have a slower absorption rate and a higher Gem plasma concentration (Fig 8C and 8D). We observed a rapid decline in Gem-TSLnps concentration to 0.5 μg/mL within 2 hr after it reached a maximum plasma concentration (C_max_) of 1.25 μg/mL. The Gem curve showed a rapid fall in concentration from C_max_ of 1.0 μg/mL to 0.4 μg/mL within just 30 min (Fig 8C and 8D). In Table 3, CL (239.06 ± 0.02 mL/hr) and V_d_ (93.64 ± 1.79 mL) of Gem was significantly greater than that of Gem-TSLnps (CL = 14.21± 0.77 mL/hr and V_d_ = 24.26 ± 0.71 mL). In contrast, the half-life (1.20 ± 0.03 hr) and AUC (35.17 ± 0.04 μghr/mL) of Gem-TSLnps was significantly greater than that of Gem alone.

**Table 3:** Pharmacokinetic profiles of Gem and Gem-TSLnps in mouse.

Parameter	Gem	Gem-TSLnps	p-value
V_d_ (mL)	93.64 ± 1.79	24.26 ± 0.71	***
CL (mL/hr)	239.06 ± 0.02	14.21 ± 0.77	***
t_1/2_(hr)	0.27 ± 0.01	1.20 ± 0.03	**
K_12_ (1/hr)	0.45 ± 0.02	0.01 ± 0.003	***
K_21_ (1/hr)	2.85 ± 0.05	0.59 ± 0.12	***
K_10_ (1/hr)	2.55 ± 0.06	0.59 ± 0.02	***
AUC (μghr/mL)	2.09 ± 0.01	35.17 ± 0.04	***
AUMC (μghr^2^/mL)	0.82 ± 0.02	60.01 ± 1.59	***
MRT (hr)	0.39 ± 0.02	1.71 ± 0.05	***

Intraperitoneal injection of free Gem 20 mg/kg, Gem-TSLnps (dose equivalent Gem 10 mg/kg)

**< 0.01

***< 0.001

ns = not significant (mean ± SD).

**Fig 9:**
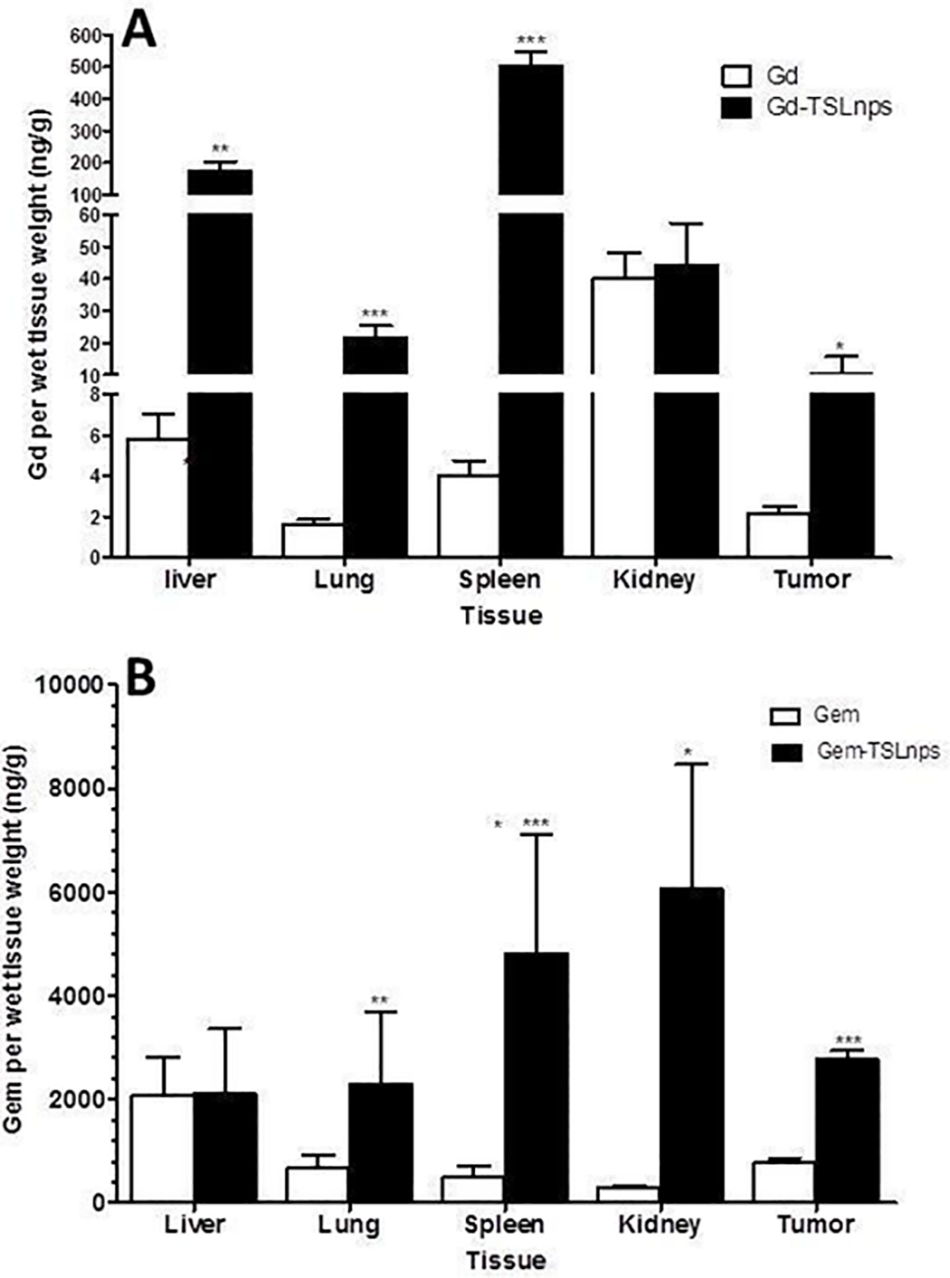
Quantitative measurement of Gd-TSLnps and Gem-TSLnps in mice organs following i.p injection of 4mg /kg of Gd or Gd-TSLnps (Gd dose equivalent, 4mg /kg); 20 mg/kg of Gem or Gem-TSLnps (Gem dose equivalent, 10 mg/kg). (A) Gd and Gd-TSLnps in tissue, (B) Gem and Gem-TSLnps in tissue. ICP-MS and HPLC were used to analyze Gd and Gem respectively. Data is expressed as mean ± S.D, n = 5 per group (p* < 0.05; p**<0.01; p***<0.001).

Accumulation of Gem through TSLnps combined with mHT after 24 hr post injection was markedly high in tumor, spleen, kidney and lung (Gem/wet tissue wt. ng/g) compared with the amount of free Gem deposited (Fig 9B). Gem amount in plasma or tissues was determined by using HPLC analysis.

#### Gd and Gd-TSLnps

The quantification of Gd in plasma and tissues was determined by ICP-MS. The Gd/Gd-TSLnps plasma concentration-time curve (Fig 8A and 8B) shows a similar biphasic behavior to that of Gem and Gem-TSLnps (Fig 8C and 8D). Gd plasma concentration peaked faster just at 15 min (0.25 h) with a C_max_ of 0.14 μg/μL compared with Gd-TSLnps with C_max_ of 0.11 μg/μL at 30 min (Fig 8A and 8B). Thereafter, Gd-TSLnps concentration declined gradually compared to that of Gd curve. Despite the same dose of Gd administered, the AUC of Gd entrapped in TSLnp was 3.9-fold high compared to free Gd after 24 hr (Table 4). Also, our results showed no significant difference in CL and V_d_ between Gd and Gd-TSLnps although CL of Gd appeared to be higher than the CL of Gd-TSLnps. As expected, TSLnps significantly increased the half-life of Gd from 0.48 ± 0.13 h to 1.39 ± 0.27 hr Table 4.

**Table 4:** Pharmacokinetic profiles of free Gd and Gd-TSLnps in mouse.

Parameter	Gd	Gd-TSLnps	p-value
V_d_ (mL)	0.20 ± 0.97	0.35 ± 0.29	ns
CL (mL/hr)	0.39 ± 0.15	0.19 ± 0.06	ns
t_1/2_(hr)	0.48 ± 0.13	1.39 ± 0.27	**
K_12_ (1/hr)	0.42 ± 0.39	0.011 ± 0.008	**
K_21_ (1/hr)	1.51 ± 0.19	0.55 ± 0.14	**
K_10_ (1/hr)	1.51 ± 0.38	0.54 ± 0.14	*
AUC (μghr/mL)	0.89 ± 0.25	3.49 ± 1.53	*
AUMC (μghr^2^/mL)	0.11 ± 0.002	4.88 ± 0.037	***
MRT (hr)	0.69 ± 0.19	1.90 ± 0.47	*

Intraperitoneal injection of Gd (Magnevist^®^) 4 mg/kg and Gd-TSLnps (Gd dose equivalent– 4 mg/kg)

*< 0.05

**< 0.01

***< 0.001

ns = not significant (Mean ± SD).

For bio-distribution of Gd and Gd-TSLnps as shown in Fig 9A, the delivery of Gd through TSLnp into liver, spleen or lung was significantly high compared with Gd alone. However, no significant difference in accumulation of Gd in the kidney was observed between Gd and Gd-TSLnps. Most importantly, 10 ng/g of Gd was deposited into tumor (Gd/wet wt. to tumor) through TSLnps which was 5.0-fold high compared to that of Gd alone accumulation in tumor (Fig 9A).

## Discussion

It has been suggested that improvement in anti-cancer drug efficacy would require an effective drug delivery system. Further, it is imperative that a reliable method for detecting tumors early will pre-inform clinicians on timely intervention leading to improved patient prognosis [7]. As such, development of an effective thermo-sensitive liposomal delivery system loaded with anticancer drug and/or contrast agent would be beneficial. First, it would improve the delivery of a high payload of anticancer drug and provide real-time assessment to determine the level of drug release at specific heated tumor sites. This would prevent the need for additional treatment caused by premature drug release at non-tumor sites resulting in insufficient drug quantity reaching the tumor. Second, it could serve as a diagnostic probe to non-invasively monitor localized tumor (early tumor) that which could momentously improve the detection and visualization of PCa. Lastly, monitoring of drug delivery provides a prediction of potential therapeutic response and may serve as a basis for future therapy planning for patients through evaluation of the tumor drug accumulation patterns for individual patients.

In this current study, we investigated the use of pegylated thermosensitive liposomal nanoparticles (TSLnps) formulation for the delivery of Gem and Gd-based contrast agent. Although Gem appeared to have a broad therapeutic spectrum against many cancers, poor cell membrane permeability and short half-life of Gem have led to long infusion time in the clinics with increased adverse effects. One of the major reasons for Gem’s instability is that Gem is attacked at its 4-amino group by cytidine deaminase to an inactive form which is rapidly eliminated from the systemic circulation [38, 39]. As part of our studies, we therefore hypothesized that anticancer activity and pharmacokinetic profile of Gem could be improved by delivering Gem directly to the tumor with TSLnps as mediated delivery system, for which Gem would be protected from enzymatic degradation. Furthermore, TSLnps due to its lipophilic nature is highly considered to positively influence the cellular uptake and internalization of Gem. To aid in early detection and visualization of tumor via MRI, we attempted to investigate the suitability of using TSLnps for the delivery of Magnevist^®^, a gadolinium-based contrast agent (Gd-TSLnps) as a possible diagnostic probe. It was our expectation that Gd-TSLnps could increase *in-vivo* stability of Gd and provide a long window of imaging tumor.

The preparation of pegylated Gem-TSLnps and Gd-TSLnps through thin film hydration method yielded 41% Gem and 62% Gd, respectively. Two strategies are possible for the encapsulation of Gem and/or Gd. One strategy is to combine Gem and Gd in two separate TSLnp delivery systems, with one loading only the anticancer drug and the other loading only the contrast agent [40]. This strategy allows a greater amount of Gem and Gd to be loaded. On the other hand, the second strategy allows for both Gem and Gd to be loaded in the same TSLnps, limiting the amount of both components in each TSLnp [41–43]. It should be noted that smaller hydrodynamic size Gd-TSLnps may be due to tight packing of Gd. The overall small size range of Gem-TSLnps and Gd-TSLnps coupled with relatively low zeta potential value might have increased their distribution in the tissues and organs [44, 45]. Furthermore, the presence of poly-(ethylene glycol) (PEG) on TSLnps surface might have created a steric barrier to prevent rapid uptake by the reticuloendothelial system and increased blood circulation time [44, 46, 47]. For long-term stability, all Gem-TSLnps and Gd-TSLnps formulations were lyophilized and stored at 4°C.

Gem-TSLnps exhibited a temperature sensitive drug release with ∼65% Gem release in less than 10 min at 42°C while less than 25% Gem was released after a period of 2 hr at 37°C. The fact that insignificant amount of Gem release was observed beyond 42°C, suggests that TSLnps phase transition temperature (T_m_) may be close to or around 42°C. To verify this, DSC was utilized to investigate the thermodynamic property of TSLnps. As expected, T_m_ of TSLnps was found to be at 41.1°C which is highly comparable to 42°C. At T_m_, TSLnp becomes disrupted, leading to increase liposomal membrane permeability and rapid release of drug through the grain boundaries [48].

To predict the mechanism of Gem release from TSLnp, regression coefficients (R-value) generated following fitting of *in-vitro* Gem release data to a number of kinetic models (Fig 2) were assessed. Based on the criteria, Gem release kinetics was observed to fit well to Korsmeyer-Peppas equation with a Fickian release exponent (n = 0.22). This suggests that Gem release from TSLnps occurs primarily through diffusion [49]. It should be noted that *in-vitro* release behavior or release kinetics of Gd was not conducted. Our previous studies in a similar study indicated that Gd linked to nanoparticle through covalent conjugation did not release any appreciable level of Gd *in-vitro* [32].

One of the most obvious factors that we strongly considered to have effect on the rheological behavior of TSLnps was temperature. TSLnp is a temperature sensitive, which implies that a relatively small variation can result in a great change in viscosity, which may change the behavior of TSLnps at 37°C and 42°C. But our rheological studies revealed no significant difference in behavior of TSLnps at room temperature, 37°C or 42°C. The rheological parameters considered were changes in consistency coefficient (k) and flow behavior index (n) at the three different temperatures (Table 2, Fig 2). Based on the data, we strongly suggested that TSLnps exhibited a Newtonian system, and the viscosity would not significantly change at these three different temperatures [27, 50].

For TSLnps to efficiently deliver anticancer drug to a locally heated tumor, the TSLnps must retain the drug in the blood circulation in stable manner before reaching the target. Based on the pharmacokinetic parameters and biodistribution data, Gem-TSLnps were determined to be significantly stable. It should be emphasized that drug dose was based on maximum deliverable concentration (dose/volume) and therefore comparison was not between equal doses of free Gem and Gem-TSLnps. Remarkably, Gem-TSPnps (10mg/kg, Gem equivalent) half the dose of free Gem (20mg/kg) was equally effective as that of free Gem in inhibiting MiaPaCa-2 subcutaneous tumor growth. We attributed the improved antitumor activity of Gem to increase systemic stability and bioavailability (AUC) by TSLnps and, the mild hyperthermia. Based on this, we speculate that Gem-TSLnps may significantly suppress subcutaneous tumor growth for equal doses of free Gem and Gem-TSLnps. After TSLnps formulation is fully optimized for higher Gem payload and temperature sensitive increased.

In this study, we employed local mild hyperthermia for the purpose of increased perfusion and enhanced vascular permeability in tumor, and increased disruption of TSLnps to release content [51]. Hence, hyperthermia enhances the delivery of Gem and improves tissue oxygenation[52, 53]. It has been previously reported that mice, treatment with thermosensitive liposomal doxorubicin and local hyperthermia results ingreater intratumor doxorubin concentration and improves therapeutic efficacy, compared with those treated with free doxorubicin[53].

To significantly enhance MRI contrast, any contrast agent carrier is expected to maintain high concentration of the agent in the desire target during the imaging time window to ensure faster and more sensitive imaging. With this in mind, we developed Gd-TSLnps and tested initially in *in-vitro*. The acquired phantoms MR images appeared to exhibit significant contrast without mild hyperthermia (Fig 5). Contrary to our *in-vivo* data, no significant difference was observed between the MRI contrast of free Gd *ex-vivo* tumor image and that of Gd-TSLnps *ex-vivo* images irrespective of time points the *ex-vivo* tumors were acquired. We conjectured that the insignificant difference in MRI contrast enhancement between free Gd and Gd-TSLnps *ex-vivo* images may be due to the: (i) fact that free Gd phantoms were not prepared, (ii) MR images of free Gd phantoms were not compared with that of Gd-TSLnps prior to *in-vivo* study and, (iii) absence of local mild hyperthermia application to the tumors after Gd-TSLnps administration. In addition, we plan to consider targeted and non-targeted temperature-sensitive liposomal carrier systems proven in literature to be more effective in enhancing MRI contrast in our future studies in real time *in-vivo* imaging. T_2_-weighted analysis was performed on *ex-vivo* tumors despite the fact that T_2_ has not been seen as a reliable analysis method due to the high vascularization of the tumor and potential T_2_ shortening due to blood residues. It is otherwise known that compartmentalized Gd (and other paramagnetic contrast agents) will show T_2_ but no T_1_ weighting due to T_1_ quenching [8].

Putting together, this study was conducted, as a proof-of-concept, to investigate the feasibility of delivering water-soluble and poorly membrane permeable anticancer drug via TSLnps delivery system. Based on this, subcutaneous tumor models were studied with specially designed heating device. It should be mentioned that these mice models were not the most representative tumor models for studying the treatment of PCa. In future studies, more realistic orthotopic tumor models combined with an image guided heating method, such as MR-guided focused ultrasound would be used.

## Conclusion

TSLnps encapsulated with poorly permeable Gem or Gd was prepared successfully. TSLnps displayed temperature sensitive drug release *in vitro* and improved PK profiles of Gem and Gd over free Gem and free Gd. Furthermore, biodistribution of Gem-TSLnps and Gd-TSLnps showed increased accumulation of Gem in the heated tumor (3.5-fold higher than free Gem, despite a significantly lower drug dose: free Gem 20 mg/kg compared with Gem-TSLnps 10 mg Gem/kg) and this increased in Gem uptake might have led to significant tumor growth inhibition of a MiaPaCa-2 tumor model. A similar trend by Gd-TSLnps was observed over free Gd. For *ex-vivo* tumor MR imaging, delivery of Gd by TSLnps appeared to show no significant contrast over free Gd. This study provides evidence to suggest that TSLnps could be used as a potential drug delivery system for the delivery of poor membrane permeable drugs. To fully evaluate the contrast enhancement capability of Gd-TSLnps, our future studies will involve optimization of TSLnps for higher Gd payload and, the comparison of real time (live) tumor and *ex-vivo* tumor MR images.

## Supporting information

S1 Table In-vitro cumulative release and kinetic models of Gem from TSLnps at 37°C and 42°C. (XLSX)

S2 Table In-vivo tumor measurements and survival data. (XLS)

S3 Table Pharmacokinetic and biodistribution data for free Gem, Gem-TSLnps free Gd and Gd-TSLnps. (XLSX)

S4 Table Rheological parameters of TSLnps at 28°C, 37°C and 42°C. (XLSX)
